# Cancer Survivors’ Experiences of Navigating the Australian Health Care System for Physical and Mental Health Care Needs

**DOI:** 10.3390/ijerph20053988

**Published:** 2023-02-23

**Authors:** Amelia Gulliver, Alyssa R. Morse, Michelle Banfield

**Affiliations:** Centre for Mental Health Research, National Centre for Epidemiology and Population Health, The Australian National University, Canberra, ACT 2600, Australia

**Keywords:** cancer survivor, health care, mental health, service access

## Abstract

People living with cancer experience many impacts on their health and mental health, and are thus likely to require ongoing health care. The aim of the current study was to investigate the health and mental health care experiences and needs of Australian cancer survivors. A total of 131 people (119 female, 12 male) with lived experience of a cancer diagnosis (at least 12 months ago) participated in an online survey collecting qualitative and quantitative data, advertised via social media groups and paid advertising. Analysis of the written responses was conducted using inductive qualitative content analysis. The findings showed that a major issue facing cancer survivors was difficulties around access to and management of services for both their mental and physical health. There was also a strong preference for increasing access to allied health care, such as physiotherapy, psychology, and remedial massage. There appear to be some inequities in the experiences of cancer survivors, particularly in accessing care. Improving the experiences of health care for physical and mental health cancer survivors should focus on increasing access to and improving the management of services, specifically allied health, through a variety of avenues, including reducing costs, increasing transport, and providing closer and more co-located services.

## 1. Introduction

Cancer is a prevalent and leading cause of disease burden globally [1,2]. Across the world, there are 44 million cancer survivors who have been diagnosed within the previous five years [3]. Australia has one of the world’s highest cancer survivor rates [4], with more than a million of these survivors living in Australia [5]. Cancer incidence has also increased sharply by 40% since 2007, with almost 150,000 Australians diagnosed in 2020 alone [6]. Significant treatment advances have led to a substantial decrease in cancer mortality in adults [7]. Cancer is now frequently considered a chronic disease, with 68% of those diagnosed expected to survive ≥ 5 years [5]. Chronic diseases are persistent conditions of a long duration (>6 months); frequently detrimental to an individual’s quality of life, they carry significant social and financial consequences [8]. However, there is a paucity of research on the needs of chronic or long-term cancer survivors globally, despite the identification of this gap a decade ago [9].

Cancer survivors typically have poorer health and mental health than those without history of cancer [10]. They frequently experience ongoing health-related sequelae, including multiple comorbid problems such as cardiovascular issues, severe side effects from ongoing treatment, and secondary cancers [11]. They typically face unique short and long-term health issues, which can affect their quality of life, family functioning, and place other demands on them, including the amount of time lost and the financial costs of frequent monitoring and tests [12]. Cancer survivors also experience high rates of mental disorders, with trauma related to the experience of cancer, fear of cancer recurrence [13], and fear of disease progression [14] being significant ongoing psychological problems that specifically affect cancer survivors [13]. In a 12-month period, 40% of cancer survivors will experience a mental disorder, which is double the rate in the general population [15,16]. Cancer survivors experience significantly higher rates of anxiety (15.8%) and depression (12.5%) and report substantially poorer mental health as late as 10 years post-diagnosis, compared to people without a history of cancer [17]. Poor mental health has a devastating effect on cancer survivors’ lives, leading to longer hospital stays [18], elevated mortality rates [19], and considerable difficulty in returning to the workforce [20].

Despite elevated and ongoing risks of psychological distress, mental health help-seeking is low. Amongst cancer survivors with diagnosable mental disorders, 90% will not seek professional help within 15 months of their cancer diagnosis [21]. Barriers to seeking mental health care for cancer survivors are similar to the general community, with some of the primary issues, including stigma and a preference for self-reliance [22,23]. Online and mobile-based therapy has been offered as a potential solution to this gap in care, partly because they can specifically address such barriers by providing private, and frequently self-guided therapy [24]. Internet and mobile-based programs have been found to be somewhat effective in improving mental health in people with cancer [25,26]. Since the advent of the COVID-19 pandemic, therapy that could be delivered distally, such as via the internet, has become clear in enabling greater numbers of people to access evidence-based mental health care [27]. Yet, few studies have examined broad scale attitudes towards online treatment in cancer survivors [28].

Appropriate follow-up health and mental health care during cancer survivorship includes screening and prevention, and care for the management of any treatment effects or chronic conditions [29]. Thus, as a chronic condition in the long-term, cancer survivors are likely to be frequent consumers of both health and mental health care, whether they are acute (first diagnosis or relapse), chronic (slow progression, or alternating remission/relapse), long-term (remission, relapse risk, or late treatment-related sequelae), or cured (disease-free survivors, life expectancy equal to general population) [30]. In Australia, government subsidized health care assists those with chronic health conditions such as cancer to access care plans that allow them to access five partially subsidized allied health services annually to help manage their condition via a Team Care Arrangement (TCA) as part of a General Practitioner (GP) Chronic Disease Management Plan. Similarly, those with mental health problems can see a GP and be referred to an allied health practitioner (e.g., psychologist, occupational therapist, social worker) for up to 10 government-subsidized sessions per calendar year using a general practitioner mental health treatment plan, previously named and more commonly known as mental health care plans.

Previous research in the US has examined health care utilization in large scale surveys of cancer survivors [31], and there have been some studies in Australia focusing on specific issues, such as shared care between cancer specialists and primary care [32], or perspectives on survivorship [33]. However, investigation of the health and mental health care needs of cancer survivors is severely lacking, particularly for those not currently in the acute phase of survivorship. 

### Aim

The current study seeks to fill the above research gap by investigating the experiences and preferences for health and mental health care in Australian cancer survivors. We also sought to investigate their mental health help-seeking attitudes, what has worked for them in the past to manage their mental health, and their ongoing needs to live well in the community.

## 2. Materials and Methods

### 2.1. Ethical Approval

The Australian National University Human Research Ethics Committee (ANU HREC protocol number 2019/739) provided ethical approval for the current study. The main ethical issues relevant to the study included ensuring voluntary participation and informed consent, providing participants with appropriate resources in the case of distress, and addressing issues of confidentiality and security in data collection and reporting. 

### 2.2. Study Design

The study design was a cross-sectional online survey using both closed and open-ended questions conducted on the Qualtrics survey platform.

### 2.3. Participants and Recruitment

A total of 142 people agreed to participate, and of these 131 (92%) completed at least one question beyond the basic demographics and were included in the study. Participants were recruited for the survey using advertising through Australian Facebook cancer support groups e.g., Cancer Warrior Australia, CML (‘Chronic Myeloid Leukaemia’) for Australians, and via a paid advertisement link on Facebook. Participants were required to self-report that they had lived experience of a cancer diagnosis (at least 12 months ago), were fluent in English, living in Australia, aged 18 years or older, and were not currently experiencing distress. The timeframe of at least 12 months from diagnosis was selected to aid in protecting potential participants who may have been in the acute stage of diagnosis and treatment, more accurately targeting our groups of interest. In addition, we provided all potential participants, including those who were not eligible, with a list cancer-specific, crisis support, and more general mental health help-seeking resources in case distress occurred. After reading a full description of the study, participants provided informed consent by ticking a box marked ‘yes’ to indicate that they had read and understood the information provided, they were eligible for the study, and agreed to participate. 

### 2.4. Survey Measures

#### 2.4.1. Demographic Information

We requested the following characteristics: gender, age category, language spoken at home, level of education, employment status, region/area of residence, diagnosis type, years since diagnosis, and finally whether they had ever in their life participated in psychological treatment for any psychological problem. We also asked if they were aware of and/or had themselves a current medical plan from the following publicly funded health plans available in Australia: (1) Chronic Disease General Practice Management Plans, and (2) a GP Mental Health Care Plans (now termed a “GP Mental Health Treatment Plan”). We also provided a link that explained the two plans to confirm meaning for participants on the Australian federal department of health website (health.gov.au). 

#### 2.4.2. Quantitative Data Collection

Quantitative questions included whether participants were offered a referral to a mental health professional at diagnosis, and whether they accepted that offer, which type of mental health professional, and the number of visits they had. We also asked about their current access to mental health care, including which mental health professionals, and participants’ satisfaction with the management of their mental health care (1 = very unsatisfied, and 5 = very satisfied). Finally, we asked them to rate their satisfaction with the management of health care for their physical health (1 = very unsatisfied, and 5 = very satisfied).

We measured participants’ attitudes to seeking professional psychological help using the short form of the Attitudes Towards Seeking Professional Psychological Help scale (ATSPPH-SF [34]). We used the 5-item scale [35] that asks respondents to rate their view on statements about psychological treatment using a 4-point Likert scale (disagree = 0, agree = 3). Scores range from 0 to 15, with higher scores indicating more positive attitudes towards seeking professional help. The 5-item scale has previously shown sound psychometric properties (*α* = 0.72) [36]. In the current study, the Cronbach’s alpha was 0.76. We also measured perceptions of online psychological therapy using 12 items from the validated Perceptions of Computerized Therapy Questionnaire-Patient Version (PCTQ-P) [37]. The PCTQ-P contains five subscales designed to measure views on online psychological therapy, each having adequate internal reliability using Cronbach’s alpha or Pearson’s *r* (2 item scales): relative advantage (*α *= 0.73; e.g., “I like that computerized therapy is anonymous”), compatibility (*α *= 0.88; e.g., “I have a positive attitude about computerized therapy”), complexity (*r* = 0.66; e.g., “It is easy to use computerized therapy”), observability (*r* = 0.56; e.g., “I have heard of computerized therapy before now”), and future use intentions (*r* = 0.72; e.g., “I plan to use computerized therapy in the future”). Items are rated on a 7-point scale (strongly disagree = 1, strongly agree = 7). These were comparable with the corresponding scales from the original PCTQ-P (*α *= 0.77–0.88, *r* = 0.42 for future use intentions) [37].

#### 2.4.3. Qualitative Data Collection

Open-ended questions to gather qualitative data for the current study included asking participants to describe their (1) experiences of accessing health care to manage their physical health, and (2) in an ideal world, how they would like to experience health care for their physical health. This was followed by the same two questions describing (3) experiences accessing mental health care, and (4) their ideal services for mental health care. We also asked (5) what has worked for them in the past in managing their own mental health, and (6) how they would describe their overall needs to live well in the community. Finally, we asked if they had (7) any further comments they wished to add.

#### 2.4.4. Data Analyses

Mean scores were calculated for help-seeking attitudes, perceptions of online therapy, and satisfaction with both physical and mental health care management. Percentages were calculated for all other quantitative responses. 

Data for the open-ended questions were coded (author AG) via a qualitative content analysis using the questions as deductive higher level codes, with themes and subthemes generated inductively under each question [38]. Data were initially coded separately under each question as this structure provided a useful a priori framework for people’s experiences, particularly as question topics included collecting both quantitative and qualitative data. Next, in the second round of analysis, co-authors (MB and ARM) identified two major cross-cutting themes of (1) Access to care and (2) Management of care. The original analysis was then revised according to these higher-level themes, and the findings were then integrated by combining the relevant quantitative data into the qualitative analysis. Data from question 5 remained separate from the two major themes, while question 6 was only partially covered by this analysis. Data were managed using NVivo 12 (QSR International).

Table 1 reports the sample demographic characteristics. The quantitative and qualitative data are presented concurrently below. Quotes are not identified by any characteristics to protect participant privacy.

Table 2 displays the quantitative data for participants’ attitudes towards and experiences of health and mental health care. For attitudes, help-seeking scores were generally positive, indicating overall participants partly agreed (mean score across items = 2.13/3.00) to the five items assessing positive help-seeking attitudes. Attitudes towards online therapy were moderately negative overall (mean score across subscales = 3.85/7.00). This was reflected in the qualitative comments as some participants remarked that computerized therapy may not be useful during this time: “A computerized therapist would not work with a human as there are facial expressions, tones of voice and feelings involved that I doubt a computer could ever evaluate”. Only one of the participants remarked that they had “not heard of computerized therapy but if it were more easily accessible it could be a great option”.

## 3. Results

### 3.1. Theme 1: Access to Care

#### 3.1.1. Physical Health Care

Table 2 demonstrates that the majority (*n* = 95; 72.5%) of respondents were currently accessing health care for their physical health at the time of the survey. The most frequently engaged health care professionals were GPs, followed by oncologist/hematologists, pharmacists, and physiotherapists. The qualitative findings around the experiences of access to health care were mixed. Approximately half of participants who responded to this question described being satisfied with their experience of access to services, although a few qualified this by saying they recognized this as a privilege via either private health cover, or being fortunate enough to be “health literate”, with some acknowledging the importance of having an ability to be “proactive” in securing their own care, with one stating that “I know that if I don’t take responsibility for my care, no-one else will”.

There were also perceived insufficiencies in the number of health appointments that could be claimed as part of a publicly funded health plan in Australia (e.g., GP Chronic Disease Management Plans). When asked about their ideal services, many responses mentioned improving access to government financial assistance for access to allied health care services (e.g., physiotherapy, massage etc.). For example, one respondent noted:

*“I would like allied health to be covered by Medicare. I get such amazing results from chiropractic care, acupuncture and massage thereby avoiding drugs of any kind and I am not a drain on the health system. Preventative medicine is my thing and allied health gives me that. But at cost!”*

Other major issues around access to health care for physical health related to barriers to care such as distance to care and transport issues, high costs, and long wait times. Some respondents suggested having more health care services located in their local area, where one person noted that having to relocate to the city away from nature was difficult: “I would rather not have had to move interstate for treatment, and to somewhere with very little opportunity to just be in nature during my initial recovery”. Many respondents suggested that health care services should be more ‘holistic’ and co-located at a single access point where “one facility would provide all required care & they would all work and communicate with each other”. Accessibility also included practical issues such as transport: “I am now 72 and had temporary blindness in one eye…transport and easy access to medical facilities, chemist and shops was my top priority”, and high costs, “I would appreciate it not being so expensive for having cancer i.e., scans and Dr bills tests etc. hospital excesses makes it an expensive disease and causes further hardship”. Finally, difficulty securing appointments with both GPs and specialists was noted as an additional problem for access due to long waiting lists. 

#### 3.1.2. Mental Health Care

Twenty-seven (20.6%) survey respondents reported currently accessing mental health care. GPs were the most frequently reported professional, followed by psychologists, and counsellors. Of those who were using mental health care, similar issues around access to physical health care were reported, with half mentioning ease of access as important, such as being referred to specialists or having care provided by their GP: “I found accessing mental health care an easy process & still visit a psychologist when I need to, via my GP & mental health plan”. However, many mentioned difficulties obtaining mental health care despite a clear need, such as prohibitive cost:

*“Many times I have chosen not to seek help for acute and chronic stress due to trying to juggle too many medical/health costs and an inability to afford sufficient numbers of appointments”.*

Some flagged a shortfall in their personal access to mental health care, citing lack of sufficient government-subsidized visits. Finally, long waiting times were also mentioned by many respondents in relation to mental health care: “having to wait too long for appointments. I…am concerned for people that need prompt treatment”. 

A total of 45 (34.4%) cancer survivors reported they were offered mental health care at diagnosis. Some participants reported they thought that it would be helpful if mental health care was offered as early as possible as “it would have been pre-emptive rather than once the problem had developed”. Many respondents noted their mental health had not been addressed at any point, with one saying “I have never been offered any mental health checks”, with another explaining: “I am 1 year 3 months in and have battled with my mind majority of that time. Yet have never been offered a mental health plan or counseling of any sort”.

In regards to those who had accessed mental health care, one participant remarked on their appreciation for the support they had received and a strong desire for others to have the same: “Help of any description should be available if needed as cancer puts you in a very vulnerable place…I grieve for those who have to go through cancer treatment alone”. Some respondents mentioned that access to ideal mental health services would feature compassionate providers, who also had some knowledge of cancer as they desired an “empathetic understanding of the vast difference between individual reaction to stress and illness that impacts on life expectancy”.

### 3.2. Theme 2: Management of Care

#### 3.2.1. Physical Health Care

Only half of cancer survivors were aware of the GP Chronic Disease Management Plans, and less than one-third reported currently having one in place. In regard to the management of their overall health care, issues primarily included difficulties with having too many specialists, or having to coordinate care themselves. One participant described it being “challenging having a lot of specialists involved in my health care, without one specialist to oversee, guide or help coordinate”. Some described a specific preference for a highly “structured” organization of care, and a health care clinic managing referrals and follow up where “help was given to make necessary appointments”. Improving follow-up practices was also considered helpful, where people felt that they “would appreciate more support assistance in between follow up scans and blood tests. Basically you’re left on your own”.

Some respondents noted that their ideal care would be with open, empathic, and knowledgeable practitioners, particularly feeling like they were supportive or that “medical people listened to me”. Many participants who described negative experiences in the management of their health care focused on issues with the professionals themselves, such as noting they had provided an incorrect diagnosis, or being “only concerned with the presenting ailment, not the root cause or any other symptoms of a bigger picture”. Some participants explained they felt their doctors were not sufficiently knowledgeable and reported challenges, e.g., “finding a GP who understands the complexities of living with a cancer diagnosis in relation to general health”.

#### 3.2.2. Mental Health Care

The majority of respondents (*n* = 92; 70.2%) were aware of GP Mental Health Treatment Plans, although less than one in five reported currently having one in place for their mental health. This may have been due to low perceived need for some participants, with some noting in the qualitative responses that they did not need any mental health services currently. However, many respondents felt that ongoing management of their mental health, particularly from practitioners they trusted, and again with an understanding of cancer and chronic illness, would have been helpful. A few respondents mentioned that frequent checks should be performed by health care providers to also identify those who may not have needed services at diagnosis, but may need them later on:

*“I think a social worker talked to me at the hospital but in general, it was left up to me… it would have been good for someone to make contact a couple of months after diagnosis to check in with how I was going mentally”.*

#### 3.2.3. Holistic Care

Whilst the questions on physical and mental health care were asked separately, many respondents noted that they would prefer mental health care to be integrated into their overall care plan, as it was critical to their ongoing recovery:

*“I am remaining strong, because of the mental health care I received, the techniques I have learned, and the knowledge that care is available, if needed, at any stage in the future”.*

Co-location was thought to be helpful by several respondents to provide “psychological care…in the same premises as all other cancer practitioners”. Management of mental health care also needs include ongoing support and follow-up from a range of practitioners, in addition to GPs, including:

*“Exercise physiologists, dieticians & psychologists who can assist cancer survivors…not only survive but thrive after treatment, with advice on what to keep an eye on, who to go to for assistance & how to recover physically & mentally so as to go on & live a fulfilling, healthy life”.*

Overall, the idea of this more holistic management of care that incorporated both mental and physical health was noted as being very important. It was also critical that this occurred over a longer time frame, with one participant remarking that “once active treatment is complete, the hard work to repair begins [with] ongoing help essential”.

### 3.3. Management of Own Mental Health

The respondents (*n* = 105) to this question identified 252 strategies total (including duplicates) they had used that they perceived as helpful in managing their own mental health. Strategies could be coded to more than one theme. An additional figure displayed in Supplementary Figure S1 shows the six themes in which responses were coded, which were the use of psychological strategies (*n* = 71), social support (*n* = 53), lifestyle strategies (*n* = 44), professional help (*n* = 40), practical strategies (*n* = 30), and other (*n* = 14).

#### 3.3.1. Psychological Strategies

These included the use of meditation or mindfulness with one participant stating that they did a mindfulness course, and that “all cancer patients should be given the opportunity as it would have been useful at time of active treatment”. Participants also employed cognitive strategies (e.g., positive thinking, acceptance, or avoidance) and reported learning more about their condition to help with their mental health: “In my case Knowledge is Power” one participant remarked.

#### 3.3.2. Social Support

Many participants noted the importance of social support. This was primarily from family and friends, with a smaller number connecting with other cancer survivors through forums or discussion groups, the latter being useful because when “speaking with people that have been through the same health issues, they have a full understanding of all issues involved”. A few people mentioned that they spent more time with pets to manage their mental health. There was also an acknowledgement from some participants that it was important “not be in isolation”. 

#### 3.3.3. Lifestyle Strategies

Exercise and keeping active physically was the top-identified physical strategy, through things, such as “doing daily yoga at home”, or other activities, such as walking, running, or going to the gym. Smaller numbers of participants identified other methods, such as a healthy diet, massage, and acupuncture, as other physical strategies that were helpful for their mental health.

#### 3.3.4. Professional Help

Most of those who listed professional sources as useful, identified mental health professionals, which were primarily psychologists. A small group identified their general practitioner as helpful in providing support for their mental health, particularly if they were monitoring their “mental health proactively”. A few people mentioned their oncologist or other specialist as providing support their mental health, with one participant remarking that they were supported by a specific oncology psychologist who helped them cope with “strong anxiety before each check up”. A few also mentioned the use of medications as being helpful.

#### 3.3.5. Practical Strategies

These included a variety of hobbies, such as music, reading, being able to attend work, staying busy, travel and planning holidays, and spending time in nature. One participant stated that the latter was the most important thing for their mental health, enjoying “above all, time spent just being in nature”.

#### 3.3.6. Other

Finally, a variety of other strategies not able to be coded to the above areas were noted, including managing their mental health by themselves, personal characteristics, such as determination, being “lucky with genetics”, or being cleared of cancer. Two participants mentioned that nothing was helpful.

### 3.4. Overall Needs to Live Well in the Community

The respondents (*n* = 98) to this question described 152 needs to live well in the community (including duplicates). Needs could be coded to more than one theme, which were social needs (*n* = 54), medical needs (*n* = 49), and basic and other needs (*n* = 49). Responses that described an overall sense of whether they felt their needs were currently being met (*n* = 25) or were not being met (*n* = 17) were also coded. An additional figure displayed in Supplementary Figure S2 shows the five themes in which responses were coded.

Social needs (*n* = 54) were similar to that covered in how people managed their mental health above, with the additional importance noted of “being actively involved in the community and being supported by the community”. Medical needs (*n* = 49) reiterated issues around access and management, and thus were included in the analysis of these two major themes above. Basic and other needs (*n* = 49) were varied, but primarily included a steady income as well as secure and safe accommodation. Basic needs noted in fewer amounts included a desire for independence, help with cleaning, and financial aid. Other needs, some of which were also coded under social needs, covered volunteering, part-time work, or helping others, and hobbies to “keep busy and focused so we don’t dwell on health issues”. Finally, some people reported that either their needs were currently being met, or that they had relatively low or simple needs to live well (*n* = 25). A smaller amount (*n* = 17) felt that there were difficulties in their life that meant their needs were not currently being met, including the experience of poor physical health, “My biggest challenge is living day to day and working with limited energy”.

## 4. Discussion

Overall, there appears to be significant variation in cancer survivors’ experiences of accessing health care for their physical and mental health. Difficulties and complexities in access to and management of services were identified across all questions regarding both physical and mental health care, as well as their needs to live well in the community.

Whilst there was considerable diversity in the experiences reported in the current study, common to both physical and mental health care, cancer survivors described significant challenges in accessing services. One prominent challenge was a high cost, including being unable to access sufficient sessions with allied health care services as part of government-subsidized services. A small number of cancer survivors specifically noted that they felt fortunate to be able to afford good-quality, private follow-up care. This is similar to previous research in the USA, which indicated that inability to afford the costs of services in cancer survivors was prohibitive to their use [31]. However, it is important to note that the USA has a substantial user pays system, which magnifies access issues, [39]. Australia’s system still includes often significant out-of-pocket costs in its fee-for-service model, reducing the potential effectiveness of chronic disease management measures [40]. A large proportion of allied health care, such as psychological services, physiotherapists, exercise physiologists, remedial massage, and occupational therapy, is delivered privately, and only partially subsidized by the Australian government, such as through a GP Mental Health Treatment Plan or Chronic Disease Management Plan with associated Team Care Arrangement [40]. Government subsidies are limited to a small number of sessions a year and commonly involve a co-payment. Thus, whilst the plans provide some measure of access to allied health, people such as cancer survivors still experience significant barriers to their ideal care. In addition, because of substantial co-payments and poor execution of plans, it is likely that some people refuse a plan, further reducing the potential reach of these measures.

Compounding the cost barriers, very few cancer survivors had a health care plan in place to facilitate access and management, despite being eligible; less than one-third had a GP Chronic Disease Management Plan, and less than one in five had a GP Mental Health Treatment Plan for their mental health. Similar to other recent research that identified the underutilization of care plans in certain groups [41], this represents a potential missed opportunity to assist cancer survivors to access additional services to assist in their long-term care. GPs were the most commonly accessed health professional for physical and mental health concerns. This critical first step in accessing broader supports through care plans could therefore easily be used to better advantage. 

Issues around management of care, such as coordinating appointments, were also mentioned as a primary issue. Some participants mentioned they found it challenging to secure and manage their own appointments across multiple services. Care coordination has been identified as challenging in Australia, partly due to the complexities of shared responsibilities for health care, where hospital care is funded and controlled via the states, and primary care is funded by the federal government [39]. In addition, managing access to multiple services has previously been highlighted as a significant problem in another chronic conditions, e.g., mental illness, where the lack of care coordination leads to treatment failure and people ‘falling through the cracks’ [42,43]. Assisting people by having dedicated care coordinators appears to be an effective strategy in providing effective wrap around care [44]. However, the lack of ongoing funding for these roles remains a persistent impediment to the provision of optimal care for chronic illness.

Cancer survivors described how they would like earlier, and more frequent access to mental health providers, particularly with those who understand the psychological impacts of cancer. Only one in five cancer survivors were accessing mental health care at the time of the survey despite help-seeking attitudes comparable to the general community [36]. This may indicate a level of unmet need given that, in a 12-month period, 40% of cancer survivors will experience a mental health problem [15,16]. However, we also do not know how many of the current survey respondents had a mental health problem that could have benefited from treatment at the time of the survey. Only one-third of cancer survivors were offered mental health support at diagnosis, and many described being unable to access or afford the psychological services they needed. It is possible that some participants were assessed for potential need, or offered a social worker or similar arrangement, and did not recognize this as mental health care. However, given the perception that some people would have liked to have been offered mental health care and did not feel they had been, it is important to make this clear. Facilitating early access to mental health care is likely to be beneficial, particularly, as respondents noted, with a mental health care provider with an understanding of the impacts of cancer.

Though few people described how they currently accessed mental health care, many more described how they would like their ideal mental health services to be, indicating a potential deficit in access to care. Cancer survivors have previously reported that psychological care is frequently neglected [39], and in some cases the need for mental health support is more important than the need for information about their cancer [45]. Thus, there may be shortfalls in the provision of appropriate psychological treatment, particularly given the high rates mental disorders experienced by this group [15,16]. Whilst computerized therapy, such as online self-help programs could improve access to mental health services, particularly where costs or distance make access difficult for cancer survivors, the acceptability of computerized services was slightly negative in this sample, with some participants expressing a preference for in-person services. However, this may have been a reflection of the age of the sample, as one of the participants, who was part of the youngest age group, expressed an interest in this type of therapy, and digital therapies typically target young people [46]. However, even though we did not specify the type of computerized therapy in the current study, some of which have greater therapist involvement (e.g., videoconferencing, therapist-guided online self-help programs), individual preferences around the care modalities should be accommodated where possible [47].

Many of the strategies that cancer survivors reported as helpful for managing their mental health were those that are frequently taught by mental health providers, such as psychologists and psychiatrists. Participants described a range of different strategies that they use to manage their mental health, which were primarily psychological techniques such as mindfulness or meditation. Previous research indicated that a high proportion of people with existing mental health problems and previous experience with therapy will use such techniques to cope during crises, and in this group these techniques may even be preventative of the development of symptoms [48]. However, people must learn them first. Thus, research to determine if early access to mental health care promotes superior mental health outcomes in cancer survivors is warranted.

### 4.1. Limitations

The current study provides significant insight into the health care experiences of a range of cancer survivors. However, there are some limitations that must be noted. Firstly, the sample was self-selected and recruited via the internet. Similar to other online research [49], we had higher rates of participation from females, which limits the generalizability of the findings. Previously, we have targeted the advertisements to increase recruitment from specific genders [36]. However, given constraints on the funding available for paid advertising, this was not feasible in the current study. We were also unable to advertise within all of the Australian support groups for different cancers on Facebook, as they reported being inundated with research requests. Thus, those with certain cancers may be under or overrepresented in the current sample. In addition, while social media recruitment provides a relatively representative community sample [49], those without access to social media are not likely to be represented by the current study. In addition, we recognize that stigma may have prevented people engaging with our study and with mental health services. The research also addressed a range of complex questions which participants were required to respond to succinctly in written format. This format allowed the collection of a greater breadth of different experiences from a large number of people, however with limited depth for each response. In the future, research would benefit from some of the key issues being pursued in greater depth in interviews or focus groups. In addition, given differences between health care service delivery and funding globally, the current study findings around service access and navigation may not be generalizable to other countries.

### 4.2. Conclusions

This study highlights significant variation in the experiences of cancer survivors in health and mental health care. However, two major cross-cutting themes around access and management regarding care were identified, and these should remain an important focus for improving both physical and mental health care for cancer survivors living in the community. Plans for the long-term health and mental health care of cancer survivors should focus on providing equitable access to holistic care, with a particular focus on the inclusion of mental health and allied health care to complement effective self-care strategies.

## Figures and Tables

**Table 1:** Demographic characteristics of participants included in the study (*N* = 131).

Characteristic,	*n* (%)
Age category (years)	
26–35	4 (3.1)
36–45	4 (3.1)
46–55	19 (14.5)
56–65	53 (40.5)
66+	51 (38.9)
Gender	
Male	12 (9.2)
Female	119 (90.8)
Highest level of education	
High school or less	41 (31.3)
Certificate/diploma	45 (34.4)
Bachelor’s degree	15 (11.5)
Postgraduate degree/diploma	29 (22.1)
Prefer not to answer	1 (0.8)
Employment	
Full-time	20 (15.3)
Part-time/casual	24 (18.3)
Unemployed	10 (7.6)
Not working (due to study, maternity leave, retirement)	47 (35.9)
Not working (due to illness or cancer treatment)	15 (11.5)
Prefer not to answer	15 (11.5)
Language	
English	127 (96.9)
English and another language	3 (2.3)
Prefer not to answer	1 (0.8)
Location	
Metropolitan	54 (41.2)
Regional	60 (45.8)
Rural/remote	17 (13.0)
Years since first diagnosed with cancer as an adult	
1–2	37 (28.2)
3–4	27 (20.6)
5–10	28 (21.4)
11+	39 (29.8)
Cancer type	
Breast	59 (45.0)
Bowel	13 (9.9)
Blood	15 (11.5)
Uterine	6 (4.6)
Ovarian	4 (3.1)
Multiple	12 (9.2)
Other * (Cervical = 3, Melanoma = 2, head and neck = 2, prostate = 2, sarcoma = 2)	20 (15.3)
Did not respond	2 (1.5)

Note: * only those with at least 2 in each category were listed.

**Table 2:** Attitudes towards and experiences of health and mental health care quantitative (*N* = 131).

Attitudes Towards Mental Health Care M (SD)			
Help-seeking attitudes (ATSPPH-SF; range 0–15; *n* = 103)	10.66 (3.33)		
Online therapy perceptions (PCTQ-P; range 1–7)			
Relative advantage (*n* = 101)	3.94 (1.27)		
Compatibility (*n* = 100)	3.54 (1.35)		
Complexity (*n* = 100)	4.43 (1.44)		
Observability (*n* = 101)	3.59 (1.81)		
Future use intentions (*n* = 100)	3.65 (1.43)		
Experiences of health and mental health care *n* (%)	Yes	No	Not sure
GP Chronic Disease Management Plan	68 (51.9)	38 (29.0)	25 (19.1)
GP Mental Health Treatment Plan	92 (70.2)	31 (23.7)	8 (6.1)
Has a plan in place			
GP Chronic Disease Management Plan	37 (28.2)	80 (61.1)	14 (10.7)
GP Mental Health Treatment Plan	25 (19.1)	102 (77.9)	4 (3.1)
Ever accessed psychological treatment in lifetime	82 (62.6)	49 (37.4)	--
	Yes	No	Can’t recall
Offered mental health care at diagnosis (*n* = 131) *	45 (34.4)	75 (57.3)	10 (7.6)
Accepted mental health care at diagnosis (*n* = 45)	27 (60.0)	17 (37.8)	1 (2.2)
	Yes	No	No answer
Currently accessing health care (physical)	95 (72.5)	34 (26.0)	2 (1.5)
Health care professional type **			
General practitioner	89 (93.7)	--	--
Oncologist/haematologist	49 (51.6)	--	--
Pharmacist	25 (26.3)	--	--
Physiotherapist	24 (25.3)	--	--
Massage therapist	17 (17.9)	--	--
Dietician	12 (12.6)	--	--
Nurse	12 (12.6)	--	--
Exercise physiologist	10 (10.5)	--	--
Acupuncturist	4 (4.2)	--	--
Occupational therapist	2 (2.1)	--	--
Other allied health *** (podiatrist = 7, chiropractor = 5)	26 (27.4)	--	--
Other specialist *** (surgeon = 12, cardiologist = 4.)	25 (26.3)	--	--
Satisfaction—physical health care management (*n* = 85; M, SD)	3.84 (1.12)	--	--
	Yes	No	No answer
Currently accessing health care (mental)	27 (20.6)	91 (69.5)	13 (9.9)
Mental health care professional type **			
General practitioner	14 (51.9)	--	--
Psychologist	14 (51.9)	--	--
Counsellor	7 (25.9)	--	--
Psychiatrist	4 (14.8)	--	--
Pharmacist	1 (3.7)	--	--
Mental health nurse	1 (3.7)	--	--
Social worker	0 (0.0)	--	--
Occupational therapist	0 (0.0)	--	--
Other allied health (“Neuro”)	1 (3.7)	--	--
Other specialist (“Art therapist, mindfulness/meditation therapist”)	1 (3.7)	--	--
Satisfaction—mental health care management (*n* = 26; M, SD)	3.77 (0.99)	--	--

Note: * Missing data (*n* = 1). ** Participants could select more than one health care professional so numbers will not sum to 100%. *** only those with at least 4 in each category were listed.

## Data Availability

The data presented in this study are available on reasonable request from the corresponding author. The data are not publicly available due to ethical constraints.

## Supplementary Materials

The following supporting information can be downloaded at: https://www.mdpi.com/article/10.3390/ijerph20053988/s1, Figure S1: Strategies that participants identified as working to manage their mental health. Larger shapes indicate a greater number of responses coded in that theme; Figure S2: Participant reported needs to live well in the community. Larger shapes indicate a greater number of responses coded in that theme.
