# Supplementary material for: Cancer Survivors’ Experiences of Navigating the Australian Health Care System for Physical and Mental Health Care Needs
Source: Int J Environ Res Public Health. 2023 Feb 23;20(5):3988. doi: 10.3390/ijerph20053988 (PMC10002190; doi:10.3390/ijerph20053988)
Supplement: Supplementary file 1 [file ijerph-20-03988-s001.zip › ijerph-2224189-supplementary.pdf]

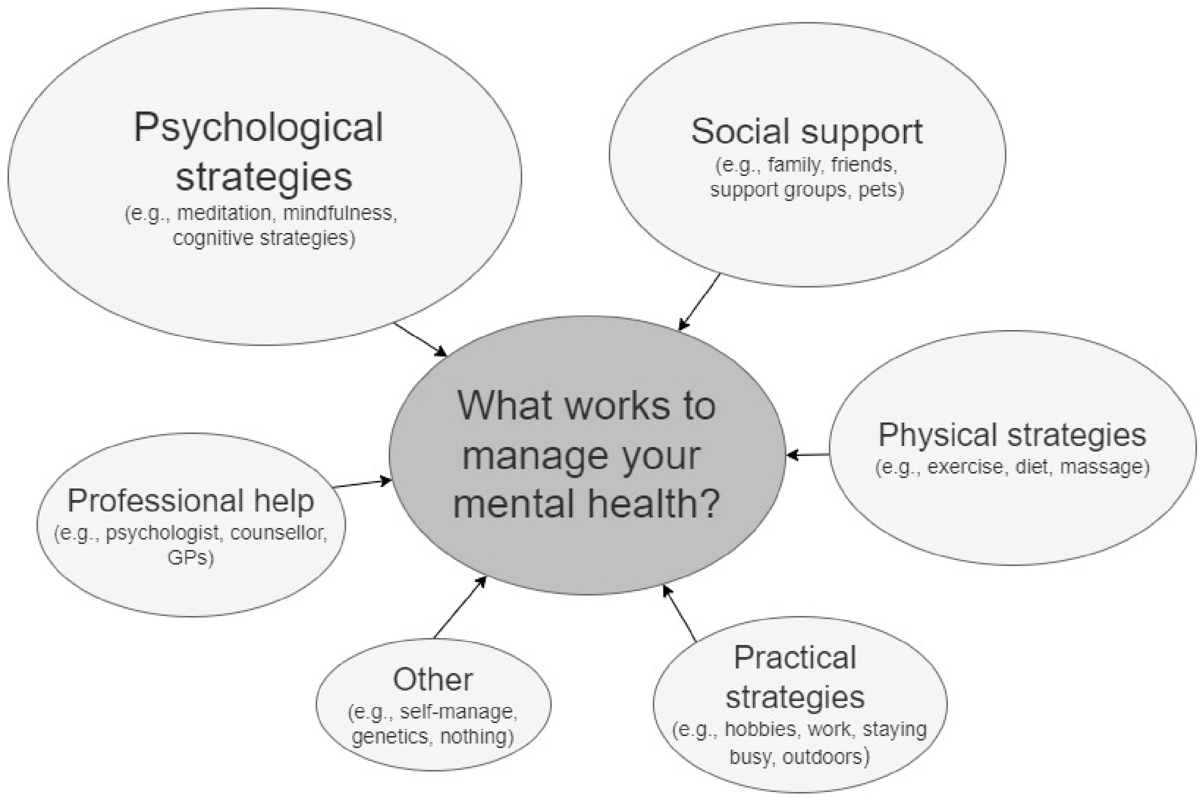


**Figure S1**. Strategies that participants identified as working to manage their mental health. Larger shapes indicate a greater number of responses coded in that theme.

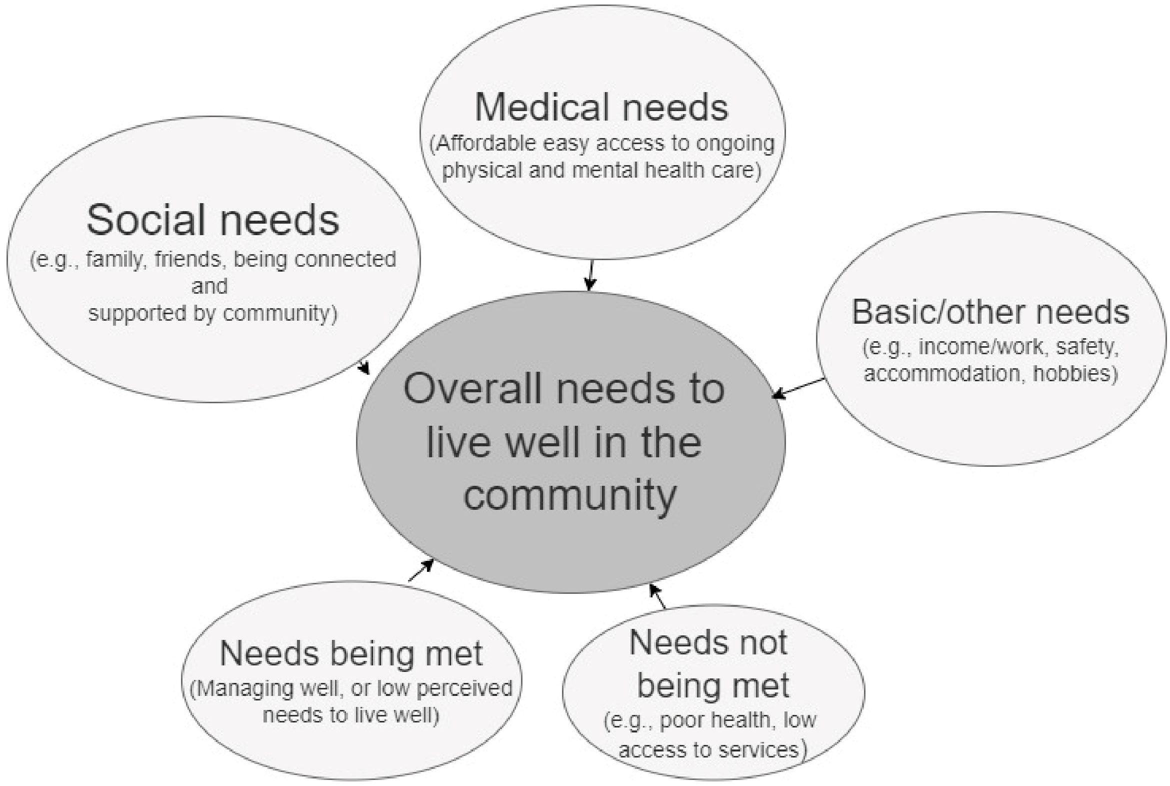


**Figure S2**. Participant reported needs to live well in the community. Larger shapes indicate a greater number of responses coded in that theme.
